# Patient experiences and discomfort associated with mid-palatal temporary skeletal anchorage devices

**DOI:** 10.1186/s40510-024-00549-9

**Published:** 2025-02-07

**Authors:** Aoife P. Barry, Vanessa Knode, Padhraig S. Fleming, Björn Ludwig

**Affiliations:** 1 School of Dental Science, Dublin Dental University Hospital, University of Dublin, Trinity College Dublin, Dublin, Ireland; 2 University Medical Center of the Johannes Gutenberg University Mainz, Mainz, Germany; 3 Queen Mary University of London, London, UK; 4 Department of Orthodontics, University of Homburg/Saar, Homburg, Saar Germany

**Keywords:** Patient experience, Patient reported outcomes, Mid-palatal, Anchorage, TSAD (Temporary skeletal anchorage device), MI (Mini-implant), OMI (Orthodontic mini-implant), Orthodontics, VAS (Visual analogue scale), Questionnaire

## Abstract

**Background:**

Mid-palatal placement of temporary skeletal anchorage devices (TSADs) has become increasingly popular among clinicians due to high success rates, low associated risk and streamlining and enhanced customization of associated supra-structures. There is however limited patient data in relation to associated experiences and impacts.

**Methods:**

A survey of patients undergoing mid-palatal TSAD insertion was undertaken using a 27-item bespoke questionnaire. Questionnaires were sent using both electronic and surface mail with a 3-month period allowed for response. Pain experience; the use of analgesia; requirement for additional visits; impacts on hygiene, speech, eating, and hobbies; and social impacts were assessed. Reponses involved the use of a Visual Analogue Scale as well as binary information.

**Results:**

Overall, 152 responses were obtained with 87.5% describing experience of TSAD insertion either “as expected” or “better”. Procedural pain was reported as mild in 62.5%. Local post-operative pain was scored as moderate in 21.1%. Some functional impairment was reported with 63.2% attributing difficulty with speech and 67.8% difficulty with eating due to the implant. However, these functional impairments were generally considered mild (by 68.1% and 60.2%, respectively) and most were very likely to recommend this treatment to others, with 65.1% (n = 99) scoring 8 or above out of 10.

**Conclusions:**

Appreciable levels of pain, discomfort and functional impairment were noted with the use of mid-palatal TSADs. However, any unpleasant experiences were generally regarded as mild with most highly likely to recommend mid-palatal TSADs to prospective patients.

## Background

The provision of temporary skeletal anchorage devices (TSADs) in orthodontics is increasingly routine being used to provide absolute anchorage without direct reliance on patient compliance. TSADs have been proven to offer a predictable means of anchorage reinforcement [1], while also providing versatility in producing a range of tooth movements in all three spatial planes.

Mid-palatal placement of TSADs may be particularly advantageous with the extra-alveolar location ensuring a reduced risk of root contact or periodontal ligament damage, while the soft tissue environment including thick keratinized mucosa is also conducive. The thick cortical bone and increased depth of palatal vertical bone height also provide good stability. The quantity and quality of bone has an effect on the success rates of orthodontic mini-implants (OMIs) [2, 3] with a cortical plate thickness of at least 1 mm associated with higher success rates [4]. Thinner cortical bone increases the dependence on the Young’s modulus of the cancellous bone with the greatest stresses and strain observed when the Young’s modulus of cancellous bone is low and the cortical bone is thin [5].

The para-median region of the hard palate at the level of the first and second premolars has been considered to be particularly suited to TSAD placement with bone depth decreasing with increasing distance from the midsagittal plane, and from anterior to posterior. Cortical plate thickness also decreases from anterior to posterior [6]. These anatomical considerations have been correlated with success rates with meta-analysis highlighting failure rates of just 1.3% in the mid-palate, with corresponding values of 16.4% for the zygomatic crest, 13.5% for mandibular buccal sites and 9.9% overall for inter-radicular placement [7].

While mid-palatal sites are increasingly seen as the site of choice for maxillary TSAD insertion, patient experiences with mid-palatal temporary skeletal anchorage devices has received little attention. Moreover, it has been shown that patient acceptance is linked to the invasiveness of orthodontic procedures [8]. The aim of this study is therefore to highlight patient experiences including pain, functional and social impacts associated with mid-palatal temporary anchorage devices.

## Methods

Ethical approval for a questionnaire-based study was obtained from School of Dental Science Research Ethical Committee, Trinity College Dublin (DSREC2023-05) and from Landesärztekammer Rheinland-Pfalz ethics committee (17095). Participants included those receiving at least one mid-palatal temporary skeletal anchorage device by a single operator in a specialist practice setting (Traben-Trarbach Germany). Participants had mini-implants placed up to August 2023 with the following selection criteria applied:

Inclusion criteria:

- Aged 10–21 years
- Non-smokers, with no allergies or medications
- No history of previous orthodontic treatment
- Willingness to participate in the study

Exclusion criteria:

- Patients aged under 10 or over 21 years
- History of orthodontic treatment

Demographic data and date of implant placement were recorded. All participants had implant placement according to the same protocol involving the use of 0.2–0.5 ml of local anaesthesia with insertion involving a guided stent and two 8 mm × 1.7 mm TSADs (OrthoEasy Pal, Forestadent, Pforzheim, Germany). All TSADs were placed para-median, 1–2 mm lateral to the mid-palatal suture at the level of the third rugae, perpendicular to the palatal bone surface, at a speed of 60 rpm, with unlimited insertion torque up to 50Ncm, using a Dentsply Sirona implant handpiece 1:20. Pre-drilling was not carried out. All clinical procedures were performed by the same orthodontist. All supra-structures were connected with the same type of abutment, attached with fixation screws (Forestadent, Pforzheim, Germany).

A 27-item bespoke questionnaires concerning intra-operative experience and post-operative recovery following the insertion of mid-palatal TSADs was developed (Appendix 1). Overall experience; pain experience and the use of analgesia; requirement for additional visits; impacts on hygiene, speech, eating, sport and other hobbies including music; and social impacts were assessed. Reponses involved the use of a 10-point VAS (Visual Analogue Scale) as well as ordinal (Much better/Better/As expected/Worse/Much worse) and binary scales. Pain was described as mild, moderate or severe with a score 0–4 is classified as mild, 5–6 moderate, and 7–10 indicative of severe pain [9, 10]. Overall, questionnaires were sent to 550 participants in January 2024. These included 319 females (58%) with 63.5% (n = 349) aged 11–14 years and just 2% (n = 11) above 18 years. A window of 3 months was allowed for responses with one reminder sent to non-responders.

## Results

A total of 152 responses were received with a response rate of 28%. Overall, 87.5% described experience of TSAD insertion “as expected” or “better”, with pain during the procedure being reported as mild in 62.5% (Table 1). Local post-operative palatal pain over the first 3 days following placement was scored as moderate in 21.1%, necessitating the use of painkillers among 33.6% over this period. More prolonged use of painkillers was required among 14.5% while 1.3% were prescribed antibiotics for associated issues. Post-operative complications necessitated an addition orthodontic appointment in 8.6% (Table 2).

**Table 1:** Experience of TSAD insertion

Experience	N = 152	%
Much Better	30	19.7
Better	55	36.2
As expected	48	31.6
Worse	14	9.2
Much Worse	5	3.3

**Table 2:** Subjective experiences associated with mid-palatal TSAD placement

	Yes	No	Total
Painkillers required over first week			
N (%)	51 (33.6%)	101 (66.5%)	152 (100%)
Painkillers required at any other time during treatment			
N (%)	22 (14.5%)	130 (85.5%)	152 (100%)
Antibiotics required for any issues during TSAD treatment			
N (%)	2 (1.3%)	150 (98.7%)	152 (100%)
Emergency visits required due to TSAD			
N (%)	13 (8.6%)	139 (91.5%)	152 (100%)
Over the course of treatment, subjectively the TSAD lead to:			
An increase in cleaning time			
N (%)	95 (62.5%)	57 (37.5%)	152 (100%)
An increase in pain on teeth			
N (%)	77 (50.7%)	75 (49.3%)	152 (100%)
An increase in pain on palate			
N (%)	98 (64.5%)	54 (35.5%)	152 (100%)
Soreness from rubbing			
N (%)	57 (37.5%)	95 (62.5%)	152 (100%)
Difficulty with speech			
N (%)	96 (63.2%)	56 (36.8%)	152 (100%)
Difficulty with eating			
N (%)	103 (67.8%)	49 (32.2%)	152 (100%)
A feeling of embarrassment			
N (%)	26 (17.1%)	126 (82.9%)	152 (100%)
Adverse effect on musical hobby			
N (%)	37 (24.3%)	115 (75.7%)	152 (100%)
Adverse effect on sporting hobby			
N (%)	32 (21%)	120 (79%)	152 (100%)

Some associated burden was noted with 62.5% noted increased time required for hygiene. Over the course of the orthodontic treatment 50.7% felt the TSAD caused more pain on teeth, although the level of discomfort was mild in 57.1%. Similarly, 64.5% and 37.5%, respectively, attributed palatal pain and pain due to rubbing during treatment to the TSAD. Again, however, associated issues were mild in 53.6% and 73.7% respectively (Tables 2, 3).

**Table 3:** Subjective experiences associated with mid-palatal TSAD

Scale	1	2	3	4	5	6	7	8	9	10	Total
Pain experienced during TSAD placement											
N (%)	24 (15.8%)	22 (14.5%)	27 (17.8%)	22 (14.5%)	15 (9.9%)	15 (9.9%)	9 (5.9%)	11 (7.2%)	2 (1.3%)	5 (3.3%)	152 (100%)
Grade		Mild 95 (62.5%)			Moderate 30 (19.7%)			Severe 27 (17.8%)			
Pain experienced over first 3 days											
N (%)	19 (12.5%)	25 (16.5%)	26 (17.1%)	14 (9.2%)	15 (9.9%)	17 (11.2%)	7 (4.6%)	14 (9.2%)	6 (4%)	9 (5.9%)	152 (100%)
Grade		Mild 84 (55.3%)			Moderate 32 (21.1%)			Severe 36 (23.7%)			
Over the course of treatment the TSAD caused:											
Pain experienced on teeth (50.66%)											
N (%)	13 (16.9%)	10 (13%)	12 (15.6%)	9 (11.7%)	12 (15.6%)	10 (13%)	6 (7.8%)	3 (3.9%)	1 (1.3%)	1 (1.3%)	77 (100%)
Grade		Mild 44 (57.1%)			Moderate 22 (28.6%)			Severe 11 (14.3%)			
Pain experienced on the palate (64.47%)											
N (%)	14 (14.4%)	8 (8.3%)	18 (18.6%)	12 (12.4%)	16 (16.5%)	14 (14.4%)	3 (3.1%)	9 (9.3%)	0 (0%)	3 (3.1%)	97 (100%)
Grade		Mild 52 (53.6%)			Moderate 30 (30.9%)			Severe 15 (15.5%)			
Soreness from rubbing (37.50%)											
N (%)	19 (33.3%)	5 (8.8%)	11 (19.3%)	7 (12.3%)	9 (15.8%)	1 (1.8%)	4 (7%)	1 (1.8%)	0 (0%)	0 (0%)	57 (100%)
Grade		Mild 42 (73.7%)			Moderate 10 (17.5%)			Severe 5 (8.8%)			
Difficulties with speech (63.16%)											
N (%)	12 (12.8%)	14 (14.9%)	16 (17%)	22 (23.4%)	10 (10.6%)	8 (8.5%)	8 (8.5%)	2 (2.1%)	1 (1.1%)	1 (1.1%)	94 (100%)
Grade		Mild 64 (68.1%)			Moderate 18 (19.2%)			Severe 12 (12.8%)			
Difficulties with eating (67.76%)											
N (%)	13 (12.6%)	11 (10.7%)	22 (21.4%)	16 (15.5%)	13 (12.6%)	9 (8.7%)	10 (9.7%)	6 (5.8%)	1 (1%)	2 (1.9%)	103 (100%)
Grade		Mild 62 (60.2%)			Moderate 22 (21.4%)			Severe 19 (18.5%)			
Adverse effects on musical hobby (24.34%)											
N (%)	22 (61.1%)	3 (8.3%)	3 (8.3%)	3 (8.3%)	2 (5.7%)	0 (0%)	3 (8.3%)	0 (0%)	0 (0%)	0 (0%)	36 (100%)
Grade		Mild 31 (86.1%)			Moderate 2 (5.6%)			Severe 3 (8.3%)			
Adverse effect on sporting hobby (21.05%)											
N (%)	25 (78.1%)	3 (9.4%)	3 (9.4%)	1 (3.1%)	0 (0%)	0 (0%)	0 (0%)	0 (0%)	0 (0%)	0 (0%)	32 (100%)
Grade		Mild 32 (100%)			Moderate 0 (0%)			Severe 0 (0%)			

In terms of functional impairment, 63.2% of patients felt the TSAD caused difficulty with speech, while 67.8% alluded to difficulty with eating. A smaller proportion (17.1%) noted feelings of embarrassment, with this generally ascribed to speech (44%) and eating (32%) issues, while appearance (16%) and hygiene (8%) implications were less frequently cited. A significant proportion (24.34%) felt that the TSAD had an impact on ability to perform music, while fewer (21.1%) cited an impact on sports (Table 3).

Overall, participants were very likely to recommend this treatment to others with 65.1% (n = 99) scoring this 8 or above (Table 4).

**Table 4:** Responses concerning likelihood to recommend mid-palatal TSADs to other prospective patients with 10 being most likely and 1 least likely (n = 152)

Response	1	2	3	4	5	6	7	8	9	10	Total
N (%)	6 (4%)	1 (0.7%)	2 (1.3%)	3 (2%)	13 (8.6%)	11 (7.2%)	17 (11.2%)	31 (20.4%)	19 (12.5%)	49 (32.2%)	152 (100%)

## Discussion

While the use of TSADs is now increasingly commonplace, lack of appropriate training, costs, fear of risk factors and scepticism about additional benefits have been considered as barriers by more than 50% of orthodontic clinicians [11]. A further barrier may relate to the dearth of patient-focussed information particularly patient experiences. This is particularly pertinent as orthodontic patients and their relatives may have associated reservations regarding mid-palatal TSAD placement as this does involve a minor surgical procedure [8].

In this study the pain experienced during the procedure of TSAD placement was relatively low, being reported as mild in 62.5%. It is accepted that young people experience physical, practical and emotional effects of orthodontic treatment [12]. Some complications with fixed appliances include sore mouth, breakages, gingivitis, with greater effects in relation to pain, speech and eating. However even with these negative effects, the literature shows that 87% would have treatment again, with 91% recommending treatment to a friend [13]. These findings correlate with the present study with 65.1% highly likely (8–10/10) to recommend mid-palatal TSAD insertion to a prospective patient. Pain is subjective and multidimensional complicating objective measurement [14, 15]. A common method used for recording or orthodontic pain is a Visual Analogue Scale (VAS) which represents a simple, valid, quick and effective tool [16, 17]. Orthodontic pain following appliance placement is felt after 2 to 4 h, peaking at 19 to 24 h, decreasing by day 3 and continues to decline until day 7 [18–21]. In our study pain in the palate over the first 3 days following placement was moderate in 21.1% with 33.6% requiring analgesia mirroring discomfort and analgesic use associated with routine therapy not involving the use of TSADs [18, 20]. In an allied study involving patients undergoing intra-alveolar buccal TSAD insertion in the maxillary second premolar to first molar region, [22] highlighted discomfort during placement, although this was low. The benefits of OMI placement were also acknowledged with up to 94.8% of participants willing to undergo treatment with OMI again [22]. The latter study, however, involved adults aged 30–50 years with TSADs inserted manually using a hand-driver.

Pain during TSAD procedures has been attributed to the infiltration of anaesthetic itself [23]. Based on previous research, patients may prefer local anaesthetic infiltration rather than the use of topical in isolation. Topical use has been linked to higher rates of failure while producing less predictable anaesthesia [24]. Sparing amounts of palatal infiltration were used in the present study with this likely to have accounted for some of the intra-operative pain reported. It is noteworthy that Pithon et al. also used a sparing amount of local anaesthesia (one-quarter cartridge), although this involved a buccal infiltration, which is typically less painful that palatal administration [22].

There is limited available data on patient experiences between different modalities of TSADs. A prospective cohort questionnaire study in Japan compared discomfort and pain after buccal and palatal TSADs as well as mini-plates [25] with the miniplate associated with more pain and discomfort. Palatal TSADs necessitated analgesic use at 12 h in 95% with miniplate, 50% buccal and 26.7% palatal insertions, respectively. No analgesics were needed with palatal TSADs after 3 days. In a further questionnaire-based survey [26] involving inter-radicular, buccal shelf, infra-zygomatic and palatal TSADs, palatal mini-implants caused highest levels of pain and caused greatest discomfort during speech and eating. These studies mirror the results of the present study with reports of more functional impairment than with alternatives, and appreciable post-operative pain although limited analgesic use was reported 3 days after insertion.

Orthodontics is associated with functional impairments including difficulty with chewing. Diet can be affected due to masticatory issues, pain, and also in response to dietary instructions [20]. Physical problems while eating may also restrict food choices [27]. It has been reported that adults are more affected by functional impairments such as mastication and potentially weight loss [28]. These findings are mirrored in the present study with 63.2% reporting speech impairment and 67.8% citing a minor degree of difficulty with eating. Feelings of embarrassment were elicited from 17.1% of patients with this typically stemming from functional impairment due to speech or eating difficulty. These observations are in keeping with experiences associated with more routine appliance therapy with Kettle et al. identifying physical, practical and emotional impacts associated with fixed and removable appliances [12].

## Limitations

Based on qualitative research, young patients do tend to adapt to functional issues attenuating any associated challenges during appliance therapy [12]. While we did examine the short-term impacts of TSADs in relation to pain experience, in particular, the longitudinal impacts of TSADs were not fully elucidated in the present study. Patient experiences are multidimensional and a questionnaire developed in one language for a population may not be appropriate for others. We therefore developed a bespoke questionnaire and implemented forward translation by bilingual experts along with back translation, accounting for cultural context in order to limit any associated bias. The response rate of 28% raises the potential for responder bias although this response rate is in keeping with similar studies. The patient demographics including healthy non-smokers, between 10 and 21 years old may affect the generalisability of the findings; however, this reflects a typical cohort undergoing orthodontic treatment.

## Conclusions

Some pain, discomfort and functional impairment were noted with the use of mid-palatal TSADs. Functional impairments chiefly included speech and eating issues, although levels of associated disability were limited. Overall, unpleasant experiences were generally considered to be low with a clear majority of patients highly likely to recommend TSADs to others.

## Data Availability

No datasets were generated or analysed during the current study.

## Abbreviations

TSAD: Temporary Skeletal Anchorage Device
PREM: Patient-Related Experience Measure
PROM: Patient-Related Outcome Measure
MI: Mini-Implant
OMI: Orthodontic Mini-Implant
VAS: Visual Analogue Scale
